# Crystal Structure of an Invasivity-Associated Domain of SdrE in *S*. *aureus*

**DOI:** 10.1371/journal.pone.0168814

**Published:** 2017-01-26

**Authors:** Miao Luo, Xiang Zhang, Shaocheng Zhang, Hongpeng Zhang, Wei Yang, Zhongliang Zhu, Ke Chen, Lei Bai, Jie Wei, Ailong Huang, Deqiang Wang

**Affiliations:** 1 Key Laboratory of Molecular Biology for Infectious Diseases (Ministry of Education), Institute for Viral Hepatitis, Department of Infectious Diseases, The Second Affiliated Hospital, Chongqing Medical University, Chongqing, PR China; 2 Department of Laboratory Medicine, People’s Hospital of YuBei District, YuBei District, Chongqing, People’s Republic of China; 3 Department of Laboratory Medicine, Chongqing Medical University, YixueYuanlu-1, Chongqing, People’s Republic of China; 4 Department of Laboratory Medicine, Hospital of Zhejiang, Xihu District, Hangzhou, People’s Republic of China; 5 School of Life Science, the University of Science and Technology of China, Hefei, Anhui, People's Republic of China; Russian Academy of Medical Sciences, RUSSIAN FEDERATION

## Abstract

The surface protein SdrE, a microbial surface components recognizing adhesive matrix molecule (MSCRAMM) family protein expressed on the surface of *Staphylococcus aureus* (*S*. *aureus*), can recognize human complement regulator Factor H and C4BP, thus making it a potentially promising vaccine candidate. In this study, SdrE^278-591^ was found to directly affect *S*. *aureus* host cell invasion. Additionally, the crystal structure of SdrE^278-591^ at a resolution of 1.25 Å was established, with the three-dimensional structure revealing N2-N3 domains which fold in a manner similar to an IgG fold. Furthermore, a putative ligand binding site located at a conserved charged groove formed by the interface between N2 and N3 domains was identified, with β2 suspected to occupy the ligand recognizing site and undergo a structural rearrangement to allow ligand binding. Overall, these findings have further contributed to the understanding of SdrE as a key factor for *S*. *aureus* invasivity and will enable a better understanding of bacterial infection processes.

## Introduction

*Staphylococcus aureus* (*S*. *aureus*) is a Gram positive opportunistic pathogen associated with the asymptomatic colonization of skin and mucosal surfaces. *S*. *aureus* is also commonly implicated in nosocomial infections, predominantly associated with catheterization or implants and community acquired infections such as wound infections, furuncles, carbuncles, abscesses, pneumonia and sepsis [1,2]. In recent years, the incidence of staphylococcal diseases has been increasing to include methicillin-resistant *S*. *aureus* (MRSA) strains, resulting in an increased infection-associated mortality. This increase has spurred efforts to develop a vaccine able to prevent these life threatening infections [3,4].

*S*. *aureus* infections rely on bacterial adhesins, which are predominantly cell wall-anchored proteins, and are grouped into a single family named microbial surface components recognizing adhesive matrix molecules (MSCRAMMs). MSCRAMMs can bind extracellular matrix proteins such as fibronectin, fibrinogen, collagen and elastin [5–8]. Serine aspartate repeat proteins (Sdr) are MSCRAMM proteins encoded by the tandemly arrayed *SdrC*, *SdrD* and *SdrE* genes located within the *sdr* locus. Sdr proteins are composed of a putative leader peptide sequence at the N-terminus, followed by an A domain and two (SdrC), three (SdrE) or five (SdrD) 110–113 residue repeated sequence. The C-terminal region of Sdr proteins contain varying numbers of the Ser-Asp dipeptides (132–170 S-D residues) followed by an LPXTG motif [7,9]. Interestingly, the three Sdr proteins are distantly related and only share 20 to 30% amino acid sequence identity, thus suggesting that these Sdr proteins have different functions in bacterial pathogenicity [10].

In a previous study, four surface-protein vaccine candidates, including IsdA, IsdB, SdrD and SdrE, were found to exhibit effective protection against a *S*. *aureus* infection alone or in combination, with the highest efficacy generated by SdrE [11]. Furthermore, one study showed that *S*. *aureus* directly binds to C4b-binding protein (C4BP), a classical complement regulator, to deter pathway-mediated bacterial opsonization and killing [12]. Additionally, SdrE can interact directly with the human major fluid-phase complement regulator Factor H, a 155 kDa plasma protein, to facilitate a staphylococcal infection [13]. Additionally, immunization with the CnaBE3 domain of SdrE was found to provide a similar efficacy as the full length SdrE protein and significantly reduced the bacterial load in mice challenged with *S*. *aureus* [10].

As a multifunctional MSCRAMM, SdrE can recognize human complement regulator Factor H and C4BP, thus potentially serving as a promising candidate vaccine. However, the structural characteristics of either full length SdrE or its functional domain remains unknown. In this study, cell infection experiments showed a similar efficacy between the SdrE N2-N3 domain and the full length SdrE, thus indicating that this domain is a key participant in host cell invasion. Furthermore, structural analysis determined the crystal structure of the N2-N3 domain SdrE^278-591^ for the first time. Similarly to the crystal structures of SdrG from *S*. *epidermidis* [14] and SdrD from *S*. *aureus* [15], the SdrE N2-N3 domain also exhibited a Dev-IgG fold [16]. Collectively, the results presented herein should shed light on the further identification of a substrate or ligand of other closely related MSCRAMM proteins and provide novel targets for the development of potent antagonists, vaccines or antibiotics.

## Materials and Methods

### Cloning, expression and purification of the SdrE

*SdrE* gene fragments corresponding to residues 141–615 and 278–591 (N2-N3 domain) were amplified using *S*. *aureus* genomic DNA as the template. The two generated PCR products were cloned into a B*am*HI/X*ho*I restriction site in a pET28a vector to generate recombinant pET28-SdrE^141-615^-His6 and pET28-SdrE^278-591^-His6 plasmids, with inserts confirmed by DNA sequencing. Transformed *E*. *coli* BL21 (DE3) were grown in LB medium at 310 K until an optical density of 0.4–0.6 (OD_600_) was reached. Next, 0.1 mM IPTG (isopropyl-β-d-thiogalactoside) was added and the temperature was lowered to 293 K and allowed to incubate overnight.

The bacterial cells were then harvested, resuspended in ice-cold lysis buffer (20 mM Tris–HCl at pH 8.0 and 300 mM NaCl) and homogenized by sonication. The cellular debris was removed by centrifugation and the resulting soluble fraction was applied to Ni^2+^-NTA affinity resin (Qiagen). After washing (40 mM imidazole, 20 mM Tris-HCl at pH 8.0 and 300 mM NaCl), the protein was eluted from the resin with 200 mM imidazole in lysis buffer. The eluent was then further purified using a DEAE Sepharose Fast Flow column (Amersham Bioscience) equilibrated with 20 mgM Tris-HCl (pH 8.0). Recombinant proteins were eluted with a linear gradient of 0–500 mM sodium chloride in 20 mM Tris-HCl (pH 8.0). Finally, fractions containing SdrE in 10 mM Tris-HCl (pH 7.0) were concentrated to about 8.6 mg L^-1^ using a Millipore Amicon concentrator with a 10 kDa cutoff membrane and the purity was determined to be approximately 90–95%, as determined by SDS-PAGE. The final purified protein was stored at 193 K for crystallization studies.

### Crystallization and structure determination

The initial SdrE crystallization conditions were determined using the setting drop vapor diffusion method by Index (Hampton Research), followed by optimization based on protein and precipitant concentration and pH. Ultimately, suitable crystals for diffraction experiments were grown within 20 days at 293 K using vapor diffusion, with 1.0 μl protein solution mixed with 1.0 μl reservoir solution and equilibrating against 200 μl reservoir solution [200 mM CaCl_2_, 30% PEG3350, 0.1 mol/L Bis-Tris (pH 6.8)]. For data collection, crystals were equilibrated against reservoir solution adjusted to 20% (v/v) ethylene glycol and then immediately placed in a 100 K nitrogen gas stream. Data were collected at 100 K on a beam line BL17U (Shanghai Synchrotron Radiation Facility, SSRF) using a MAR225 CCD detector (MAR Research). The data were processed and scaled to 1.25 Å using Mosflm software. The structure was determined using molecular replacement methods, with the *S*. *aureus* Bbp structure used as a searching model and the PHENIX packages [17] used for further refinement. Data collection and structure statistic are summarized in Table 1 and the atomic coordinates and structure factors (PDB code 5IHW) were deposited in the Protein Data Bank.

**Table 1:** Data collection and refinement statistics.

Parameter	SdrE
Space group	P1
Unit cell dimensions	
a, b, c (Å)	39.26, 41.56, 51.14
α, β, γ (°)	95.59, 97.55, 99.55
Resolution (Å)	50.32–1.25 (1.32–1.25)
Number of reflections (unique) ^a^	278044 (40094)
Completeness (%)^a^	87.0 (87.0)
Rmerge (%)^a^^,^^b^	5.6 (33.5)
R refinement	
R-value (working set)^c^	14.33
Rfree (test set, 5.1%)^c^	17.64
Number of atoms	2679
Protein	2546
Water	133
r.m.s.d.	
Bond length (Å)	0.025
Bond angles (°)	2.263
B-factors (Å^2^)	
All atoms	17.096

^a^ Values in parentheses are for the highest-resolution shells (1.32–1.25 Å)

^b^ Rmerge = ∑(I−<I>)/∑I.

^c^ Values in parentheses are for the highest-resolution shells (1.32–1.25 Å)

^d^ R-value = ∑(|Fobs|−|Fcalc|)/∑|Fobs|.

### Construction of bacterial strains

To construct Δ*SdrE* and Δ*SdrE*^*278-591*^ strains from *S*. *aureus* MU50 (ATCC 700699), *SdrE* and *SdrE*^*278-591*^ upstream and downstream regions were amplified from *S*. *aureus* MU50 genomic DNA using their specific PCR primers. The Chloromycetin (*chl*) cassette was amplified from Rosetta-gami2 (DE3) DNA by PCR. Overlap extension was performed to generate Up-chl-down (Δ*SdrE*) and Up-Chl-SdrE-N-down (Δ*SdrE*^*278-591*^) fusion fragments. Then, the overlap fragments were subsequently subcloned into a pBT2 vector to generate pBT-Δ*SdrE* and pBT-Δ*SdrE*^*278-591*^ plasmids, respectively. Bacterial cells were transformed *via* electroporation, with *S*. *aureus* RN4220 initially transformed for modification, and subsequently the modified plasmids were extracted. Subsequently, the resulting plasmids were used to transform *S*. *ureus* MU50 *via* electroporation through homologous recombination to delete *SdrE* and Δ*SdrE*^*278-591*,^ respectively. *Chl* insertions at the loci of *SdrE* were confirmed by DNA sequencing using the PCR products from genomic sequences harvested from Δ*SdrE* and Δ*SdrE*^*278-591*^ *S*. *aureus* MU50 colonies.

### Adhesion and invasion assays using human 143B and HeLa Cells

Adhesive and invasive abilities were examined as previously described with a minor modification [18]. To examine adhesivity, Human 143B and HeLa cells were inoculated and grown to confluence in 24-well tissue culture plates up to 1.5×10^5^ CFU ml^-1^ and washed three times with PBS buffer (phosphate buffer saline). Exponential-phase cultures of *S*. *aureus* Mu50 and its isogenic *SdrE* and *SdrE*^*278-591*^ mutant derivatives (10^8^ CFU) were diluted with either DMEM (for 143B cells) or RPMI 1640 (for HeLa cells) medium supplemented with 10% FBS. Host cells were then inoculated at a multiplicity of infection (MOI) of 10 bacteria/cell. After an additional 1-h incubation at 37°C, the culture media was removed from each well and the monolayers were washed three times with PBS. The cells were lysed by adding 20 μl PBS and smeared onto clean glass slide. The resuspension cells were then stained with Wright's following fixation. *S*. *aureus* concentrations were determined microscopically and represent the average of three wells from at least three separate experiments.

To assess invasivity, *S*. *aureus* Mu50 wild-type, *SdrE* and *SdrE*^*278-591*^ deficient strains were grown to 10^8^ CFU, washed and diluted in the appropriate medium supplemented with 10% FBS as described above, to include a MOI of approximately 10 bacteria/host cell. Bacterial suspensions were added to each of the cell lines and allowed to incubate for 2 h, followed by washing with PBS. Fresh medium containing 10 mg ml^-1^ penicillin and 10 mg ml^-1^ streptomycin was then added to each well in order to kill all extracellular bacteria. After an additional 1 h of incubation, the cells were then detached from the plates using 100 μl of 0.1% trypsin. The number of viable bacteria released from the cells was assessed after serially diluting the lysates on agar plates. These experiments were performed in triplicate and repeated twice.

## Results and Discussion

### *SdrE* and *SdrE*^*278-591*^participate in *S*. *aureus* host cell invasion

**Fig 1:**
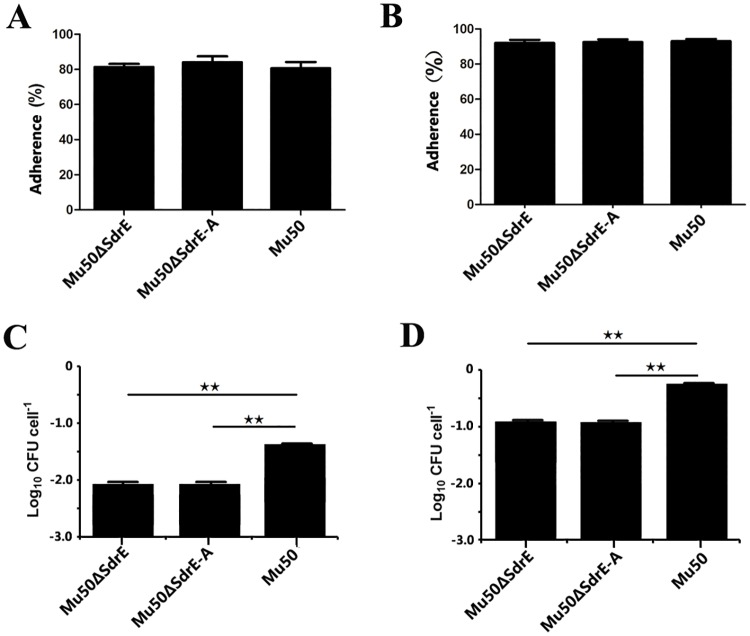
Adherence and invasion of the ΔSdrE and ΔSdrE^278-591^ mutants in host cell lines *in vitro*. *S*. *aureus* Mu50 and its isogenic mutants *ΔSdrE* (Mu50Δ*SdrE*) and ΔSdrE^278-591^ (Mu50Δ*SdrE-A*) were examined for adherence in HeLa (A) and 143B cells (B). These same mutants were also examined for invasivity in HeLa (C) and 143B (D) cells. Infectivity assessments were conducted for 4 h at 37°C. Wild-type *S*. *aureus* and the *ΔSdrE* (Mu50*ΔSdrE*) and *ΔSdrE*^278-591^(Mu50*ΔSdrE-A*) mutants were generated to be devoid of SdrE in order to avoid destruction of the monolayer infection system. Scoring of the number of adherent and invasive bacterial cells indicate that adhesion and invasion are substantially reduced for *ΔSdrE* ((Mu50*ΔSdrE*) and *ΔSdrE*^278-591^ -deficient (Mu50*ΔSdrE-A*) *S*. *aureus* mutant. Results are presented as a mean ± standard deviation for at least three independent experiments. Asterisks and triangles denote values significantly different from the wild-type as determined by Student’s t-test (** P < 0.01).

Host cell adherence and invasion are thought to be key events during bacterial infection [8]. To assess whether *SdrE* and *SdrE*^*278-591*^ are specifically involved in these processes, an in-frame allelic replacement of *SdrE* and *SdrE*^*278-591*^ in the unencapsulated *S*. *aureus Mu50* strain was performed to generate the isogenic mutants Mu50-*ΔSdrE* and Mu50-*Δ*S*drE*^*278-591*^. Using these strains, a quantitative adherence and an invasion assay were performed using 143B and HeLa host cell lines. Both the Mu50-*ΔSdrE* and Mu50-*Δ*S*drE*^*278-591*^ mutants exhibited a similar hypo-adherence phenotype compared to the wild-type strain in both cell lines (P > 0.05; Fig 1A & 1B), thus indicating that *SdrE* and *SdrE*^*278-591*^ do not inhibit *S*. *aureus* host cell adherence. However, both cell lines showed significantly attenuated invasivity when inoculated with the Mu50-*ΔSdrE* and Mu50-*Δ*S*drE*^*278-591*^ deficient strains compared to the wild-type strain (Fig 1C and 1D). To confirm this finding, a functional back mutant was generated by complementation with the mutants generated via long flanking homology polymerase reaction mutagenesis to generate the entire *SdrE* gene. Indeed, reintroducing *SdrE* and *SdrE*^*278-591*^ back into the mutant restored the invasive ability. Taken together, these results clearly show that both *SdrE* and *SdrE*^*278-591*^ contribute to *S*. *aureus* pathogenicity, with *SdrE*^*278-591*^ potentially able to recognize host membrane proteins and aid in host cell invasion.

### SdrE^278-591^ structure and model quality

SdrE^278-591^ was initially crystallized by hanging drop vapor diffusion, with crystals that diffracted to a resolution 1.25 Å obtained after 20 days. However, when subsequently calculating electron density, only SdrE^278-591^ could be clearly defined. Additionally, SDS-PAGE gel experiments confirmed that the crystal structure was a protein fragment with a molecular weight of 44 kDa, with about a 16 kDa segment degraded (unpublished data). These findings suggest that the SdrE^141-278^ region is much more flexible and more susceptible to proteolytic cleavage than the SdrE^278-591^ region.

**Fig 2:**
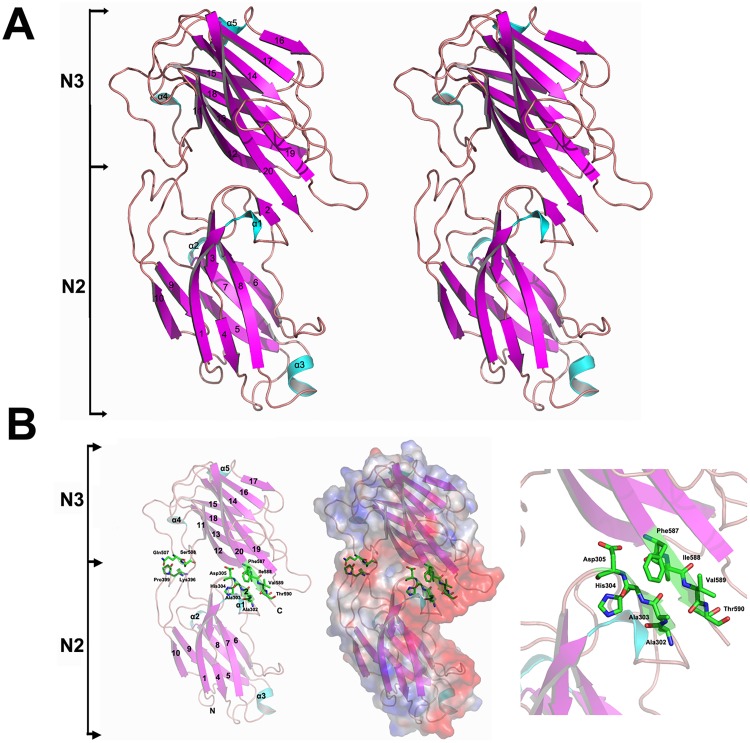
The overall structure of *S*. *aureus* SdrE^278-591^. (A) The structure of *S*. *aureus* SdrE^278-591^ in stereo view and the (B) electrostatic potential surface. A saturated red color indicates Ø<-10 kiloteslas/e and a saturated blue indicates Ø>10 kiloteslas/e; T = 293 K. The magenta arrow points towards the negatively charged groove and the sticks represent the residues in β2 and β20 directly participating in the interface between the N2 and N3 domains.

The X-ray structure of SdrE^278-591^ was determined by molecular replacement and refined against diffraction data to 1.25 Å with a 17.64% free R-factor and 14.33% crystallographic R factor (Fig 2A). The final model contained 227 amino acids (residues 278–591), 8 ethylene glycol and 540 water molecules. Furthermore, residues 592-NDTGGGDGT VKPEEKLYKIGDYVW-615 were invisible in the electron density, possibly due to a conformational disorder, and therefore were not included in the final model. The root-mean-square deviations (rmsd) from an ideal bond length and bond angle were 0.025 Å and 2.263°, respectively. In the final model, 97.99% of the residues had main-chain torsion angles in the most favored region of the Ramachandran diagram, while the remaining 2.01% of residues occupied additionally allowed regions (Table 1).

### Structural overview of SdrE^278-591^

The SdrE^278-591^ region folds into two distinct domains, the N2 and N3 domains, both of which have two layers of β-sheets and are structurally similar to a Dev-IgG fold (Fig 2A). The two β-sheets of the N2 domain are composed of 1, 3, 4 and 8 strands on one side and 5, 6, 7, 9 and 10 strands on the opposite side. In the N3 domain, 11, 12, 13, 15 and 18 strands form one principal sheet and 14, 16, 17, 19 and 20 contribute to the facing sheet. The α1 helix and two β-sheets comprising the N2 domain are located at the negatively charged groove pocket formed by N2 and N3 domains. In this region, there is a linker (423-YQD-425) and several hydrogen bonds formed between β2-β20 (Ala302-Thr590, Ala303-Val589, His304-Ile588, Asp305-Phe587) and two pairs of residues in the loop (Lys396-Ser508, Pro399-Gln507), which directly stabilizes the spatial pattern between N2 and N3 domain (Fig 2B).

There was only one SdrE^278-591^ molecule in one asymmetric unit and the crystallographic interface between the bacterial molecules showed little interaction. Furthermore, size exclusion chromatography experiments confirmed the presence of SdrE^278-591^ as a monomer in solution (data not shown). These data suggest that the monomer of SdrE^278-591^ may be a stable and functional unit in solution.

The electrostatic potential surface of SdrE^278-591^ displays prominent asymmetry. At the interface of the N2 and N3 domains, a negatively charged grove is formed that is comprised of 27 amino acids (S1 Fig), to include Asp295, Asp298, Asn299, Asp305, Asp308, 310Glu, Ser338, Asp339, Thr341, Asp342, Asp345, Asp348, Thr350, Asp351, Gln424, Asp425, Gln435, Ser436, Glu443, Asp444, Thr557, Ser558, Asp559, Glu561, Ser585, Thr590 and Ser591, with 15 of the residues conserved among various bacterial species (S1 Fig). Interestingly, the ligand binding sites of these MSCRAMM are similarly located at the charged groove, suggesting that this charged interface may be a feature of SdrE homologs enabling ligand recognition.

**Fig 3:**
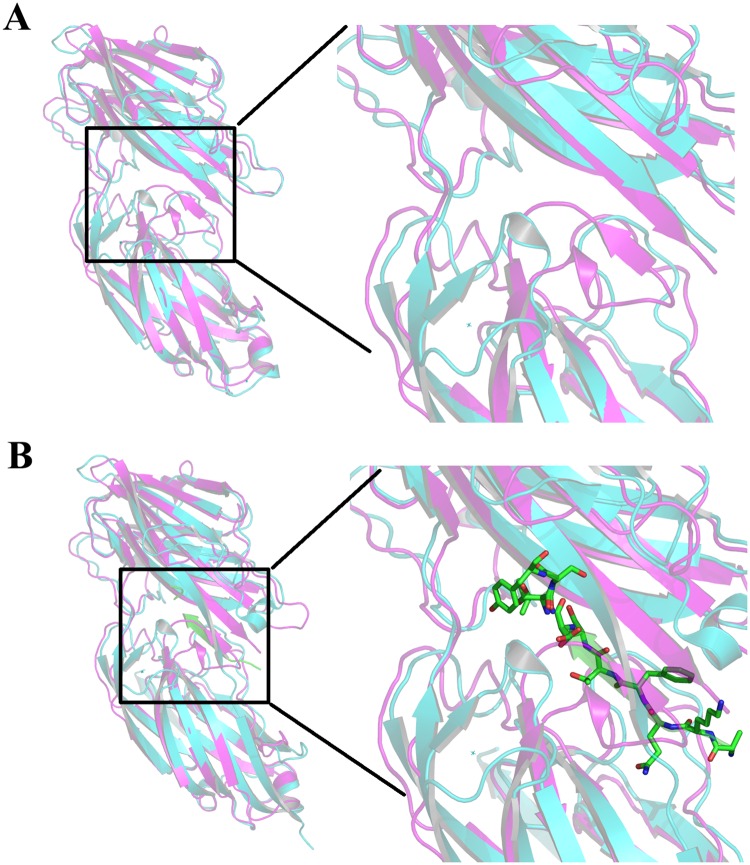
*S*. *aureus* SdrE^278-591^ (magenta) superimposed on its homolog *S*. *aureus* Bbp (cyan). (A) *S*. *aureus* SdrE^278-591^ (magenta) superimposed on its homolog Bbp (substrate free, PDB code 5cf3). (B) *S*. *aureus* SdrE^278-591^ (magenta) superimposed on its homolog Bbp (substrate, PDB code 5cfa). The predominantly green strand is the peptide ligand (substrate).

To assess the degree of the structural similarity, the SdrE^278-591^ structure was submitted to the DALI server to identity proteins with structural homologies [19]. The proteins with the highest homologies belonged to the MSCRAMM family and included bone sialoprotein-binding protein (Bbp; PDB code: 5cf3), fibrinogen-binding protein SdrG (PDB code: 1R17) and ser-asp rich fibrinogen/bone sialoprotein-binding SdrD (pdb code: 4JE0). Of these, Bbp folding was the most similar to SdrE^278-591^, with a rmsd of 2.2 Å (Fig 3A & 3B), despite several structural differences existing between these two similar structures. Compared to the structure of the Bbp loop, residues 296–306 of the SdrE N2 domain form an inserted α1 helix and two β-strands between β1 and β3. Furthermore, it is rather remarkable that β2 settled near to the β20 (A3 domain), thus functioning as a major force restricting the N2 and N3 domains via hydrogen-bond interactions, while the Bbp loop region cannot form these interactions. Additionally, residues 319–326 fold into a β-strand which functions as the linker between the N2 and N3 domains, while the corresponding residues (595–615) are missing from the SdrE^278-591^ structure.

### A putative SdrE^278-591^ ligand binding site

In a previous study, SdrE was found to be able to recognize human Factor H and participate in staphylococcal renal infections, thus making it a candidate vaccine target to aid in protection against *S*. *aureus* infection [13]. As a key virulence factor of *S*. *aureus*, the N2-N3 domains of SdrE, similarly to full length SdrE, directly participate in the invasion step during pathogen infection. While this finding implies that the functional domain of SdrE^278-591^ interacts with host membrane proteins to invade the cytoplasm, little structural information regarding interactions between SdrE and host proteins are characterized. Furthermore, the sequences and functions of MSCRAMMs, including SdrE, SdrD, SdrC and Bbp, are evolutionarily conserved, thus implying that MSCRAMMs may have arisen from a common ancestor, as indicated by their amino acids sequence homologies and similar modular organization [16]. The ligand binding activity of most of these MSCRAMMs is localized to the N-terminal regions and comprises ~500 amino acids [14]. Three MSCRAMM N2-N3 domain structures of have been determined, including Bbp from *Saureus* (PDB code: 5cf3), SdrD from *S*. *aureus* (PDB code: 4jdz), Pfba from *S*.*pneumoniae* (PDB code: 3ZPP) and SdrG from *S*. *epidermidis* (PDB code: 2RAl, 1R17). According to these determined structures, the ligand binding sites are all located at the negatively charged grooves formed by the adjacent interface of the N2 and N3 domains. In Bbp, fibrinogen α^561−575^ was found to bind Bbp^273−598^ at the open groove between the N2 and N3 domains. Additionally, the previous study found that ligand binding resulted in a rearrangement of the C-terminal and four additional regions within Bbp^273-598^ to “latch” the ligand binding site and stabilize the overall structure [20]. Similarly to that of Bbp, the substrate binding sites for SdrG and SdrD are also located at the charged groove formed by the interface of the N2 and N3 domains (Fig 3A & 3B). Accordingly, SdrE, as a MSCRAMM member, presumably possesses a putative ligand binding site located at the charged groove and formed by the interface of N2 and N3 domain. Nevertheless, when comparing the SdrE^278-591^ structure, the ligand binding site is shielded by the β2 sheet, thus resembling the ligand free structure of Bbp, SdrG and SdrC. Consequently, during ligand binding, the residues located at the interface might allosteric rearrange and the β2-sheet might disentangle from the β20-sheet and open the binding groove, thus enabling the ligand to bind the charged site until the β2-sheet returns to block the binding site. However, this speculation regarding a potential conformational change in the binding site requires further examination, to include characterizing the SdrE^278-591^–ligand complex structure.

Altogether, these findings aid in furthering the understanding of SdrE as a key factor for *S*. *aureus* invasivity. Additionally, this study reported the three dimensional structure of SdrE^278-591^, to include the N2-N3 domains, which forms a fold similar to an IgG fold, with a similar fold seen in Bbp, SdrG and SdrD. Additionally, the putative ligand binding site was located at a conserved charged groove formed by the interface between N2 and N3 domains. Altogether, these results revealed the structural basis for SdrE-ligand interactions and further contribute to the understanding of *S*. *aureus* invasivity.

## Supporting Information

S1 Fig Structure-based sequence alignment of SdrE^278-591^ with its homologs. Structure-based sequence alignment of SdrE^278-591^ with its homologs. SdrE^278-591^ from *S*. *aureus* Mu50; sialoprotein-binding protein (Bbp) from *S*. *aureus* (PDB code 5cf3), fibrinogen-binding protein SdrG from *S*. *epidermidis* (PDB code 1r17), and ser-asp rich fibrinogen/bone sialoprotein-binding SdrD from *S*. *aureus* (PDB code 4je0). The secondary structure and residue numbering for SdrE^278-591^ are shown above its sequence. Arrows represent β-strands and large coils indicate α-helices, while fully conserved residues are indicated by white letters on black back-ground and conservatively substituted residues are indicated as black letters in black boxes. The image was generated using ESPript, with secondary structure elements assigned based on 5IHW for SdrE^278-591^.
(DOCX)
