# Supplementary material for: Crystal Structure of an Invasivity-Associated Domain of SdrE in S. aureus
Source: PLoS One. 2017 Jan 26;12(1):e0168814. doi: 10.1371/journal.pone.0168814 (PMC5268492; doi:10.1371/journal.pone.0168814)
Supplement: S1 Fig — Structure-based sequence alignment of SdrE278-591 with its homologs. SdrE278-591 from S. aureus Mu50; sialoprotein-binding protein (Bbp) from S. aureus (PDB code 5cf3), fibrinogen-binding protein SdrG from S. epidermidis (PDB code 1r17), and ser-asp rich fibrinogen/bone sialoprotein-binding SdrD from S. aureus (PDB code 4je0). The secondary structure and residue numbering for SdrE278-591 are shown above its sequence. Arrows represent β-strands and large coils indicate α-helices, while fully conserved residues are indicated by white letters on black back-ground and conservatively substituted residues are indicated as black letters in black boxes. The image was generated using ESPript, with secondary structure elements assigned based on 5IHW for SdrE278-591. (DOCX) [file pone.0168814.s001.docx]

**Supporting Information**

**Structure-based sequence alignment of SdrE^278-591^ with its homologs**

Legend

S Figure. Structure-based sequence alignment of SdrE^278-591^ with its homologs. SdrE^278-591^ from *S. aureus* Mu50; sialoprotein-binding protein (Bbp) from *S. aureus* (PDB code 5cf3), fibrinogen-binding protein SdrG from *S. epidermidis* (PDB code 1r17), and ser-asp rich fibrinogen/bone sialoprotein-binding SdrD from *S. aureus* (PDB code 4je0). The secondary structure and residue numbering for SdrE^278-591^ are shown above its sequence. Arrows represent β-strands and large coils indicate α-helices, while fully conserved residues are indicated by white letters on black back-ground and conservatively substituted residues are indicated as black letters in black boxes. The image was generated using ESPript, with secondary structure elements assigned based on 5IHW for SdrE^278-591^.

**
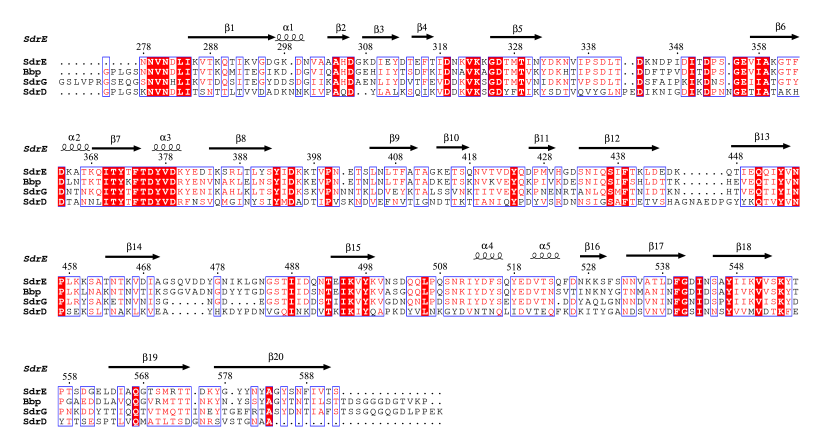
**
